# Supplementary material for: Identifying predictors and assessing causal effect on hypertension risk among adults using Double Machine Learning models: Insights from Bangladesh Demographic and Health Survey
Source: PLoS Comput Biol. 2025 Jul 2;21(7):e1013211. doi: 10.1371/journal.pcbi.1013211 (PMC12338952; doi:10.1371/journal.pcbi.1013211)
Supplement: Attachment — Submitted filename: Review_PLoS Comp Biol_doubleML.docx [file pcbi.1013211.s001.docx]

The manuscript, "Identifying Predictors and Assessing Causal Effects on Hypertension Risk Among Adults Using Double Machine Learning Models: Insights from the Bangladesh Demographic and Health Survey," thoroughly examines the factors influencing hypertension and their causal effects. Notably, the authors demonstrate that BMI and self-awareness significantly impact hypertension, with a higher BMI increasing the likelihood of hypertension. However, the results would benefit from further validation and clarification of key points.

**Major**

1. The study primarily employs the **double/de-biased machine learning model**, yet its methodological details are insufficiently explained. Given that causal inference from this approach is central to the study’s conclusions, a more detailed explanation of the **double/de-biased machine learning process** would strengthen the study’s reliability. For example, please include (1) the basic methodological concept of double/de-biased machine learning model introduced in ref (36) and (2) the performance of that model in previous studies.
2. The model performance in predicting hypertension risk was assessed by randomly splitting the dataset into training and test sets. This randomness introduces the possibility that different random seeds could yield varying prediction errors. To ensure robustness, we recommend the following validation procedure

- Perform 10,000 iterations (or a sufficiently large number) of train-test splits.
- Compute performance metrics (e.g., accuracy, error, precision) for each split.
- Report the distribution of these metrics or representative statistics such as mean, median, and standard deviation.

**Minor**

1. Figure 2: In line 208, the training and validation errors for the random forest model are reported as 0.03% and 0.65%, respectively, suggesting an accuracy (i.e. 100% – error (%)) exceeding 99%. However, the confusion matrix in Figure 2 indicates an accuracy of 93%. Could the authors clarify the reason for this discrepancy?
2. Figure 3: Feature importance can be quantified using different measures, such as frequency (or weight), gain, or total gain. Which measure was used in Figure 3?
