# Supplementary material for: Identifying predictors and assessing causal effect on hypertension risk among adults using Double Machine Learning models: Insights from Bangladesh Demographic and Health Survey
Source: PLoS Comput Biol. 2025 Jul 2;21(7):e1013211. doi: 10.1371/journal.pcbi.1013211 (PMC12338952; doi:10.1371/journal.pcbi.1013211)
Supplement: Attachment — Submitted filename: Review_Report.docx [file pcbi.1013211.s002.docx]

**General Comments**

This manuscript addresses an important topic by applying double machine learning methods to assess hypertension risk using data from the Bangladesh Demographic and Health Survey. The approach is timely, and nationally representative data is a strength. However, key aspects of the study design require clarification to ensure the findings are robust and actionable. Specific major and minor comments are outlined below.

**Major Comments**

1. The manuscript would benefit from a clearer discussion of its novelty. A related study by Islam et al. (2022, Frontiers in Cardiovascular Medicine, DOI: 10.3389/fcvm.2022.839379) used machine learning to predict hypertension using the same BDHS dataset. I recommend briefly contrasting your causal inference approach with their predictive modeling, highlighting the specific advantages of DML and your unique contribution.
2. The Methods section requires more detail to ensure reproducibility. Please specify the hyperparameters used for the Random Forest model (e.g., number of trees) and how SMOTE was tuned. Additionally, clarify the implementation of the Double Machine Learning approach, including cross-fitting, nuisance function estimation, and the software or packages used. Finally, describe how the SHAP and ICE analyses were performed and consider including a summary or appendix presenting these results.
3. The causal effect reported (OR = 1.30) is compelling but requires stronger validation. I recommend discussing the potential impact of unmeasured confounders, such as diet, and conducting a sensitivity analysis to assess the robustness of the findings. Additionally, the causal structure presented in Figure 12 should be more clearly justified with supporting evidence.
4. The manuscript would benefit from discussing the computational complexity of the methods used, particularly SHAP, ICE, DML, and Random Forest, in the context of the dataset size (11,815 samples). Please comment on runtime, time complexity, and scalability of the approach. Including a brief discussion, table, or subsection would provide valuable context for the methods' feasibility and broader applicability.

**Minor Comments**

1. The manuscript would benefit from an extensive writing and language review. There are multiple issues throughout the text, including missing or extra punctuation (e.g., extra period in line 66), subject-verb agreement errors (e.g., unclear sentence spanning lines 71–73), inconsistent terminology ("DML" versus "double/de-biased machine learning"), awkward phrasing (e.g., the "high accuracy" statement in Section 4), and incomplete figure captions (Figures 5–11 need more details on the caption). These examples illustrate broader problems, and a comprehensive revision for grammar, clarity, consistency, and formatting is strongly recommended.
2. The reported 93% accuracy is promising but would benefit from qualification. Please consider comparing your model’s performance with that of Islam et al. (2022, 90% accuracy) using a formal statistical test. Additionally, avoid strong claims such as "high accuracy" without external validation or more extensive cross-validation, and discuss the generalizability of your findings.
3. The literature review relies heavily on older references, many of which are nearly 20 years old. While some historical context can be useful, I encourage the authors to incorporate more recent studies relevant to hypertension risk prediction, causal inference, and machine learning methods.
4. The manuscript mentions that previous surveys are "not robust," but it does not clearly explain these studies' specific flaws or limitations. I recommend that the authors elaborate on what "not robust" means and provide a more detailed critique of existing approaches.
5. If any preprocessing steps were applied to the dataset, I encourage the authors to describe them clearly and, if possible, share the processed data, preprocessing parameters, and related code. Using a repository such as GitHub to make the data pipeline and code publicly available would greatly enhance the reproducibility, transparency, and scalability of the study.
6. The Decision Tree presented in Figure 4 is difficult to read due to low image quality. I recommend improving the resolution of the figure or redesigning the tree structure to enhance readability. Providing a high-resolution version or an alternative layout would make the visualization more effective and accessible to readers.
7. There are inconsistencies in font usage throughout the manuscript, with a mix of fonts such as Times New Roman, Aptos, and Arial. I recommend standardizing the font across the entire document to maintain a professional and cohesive presentation.
8. The Future Work section is too general. I recommend being more specific about potential future directions, such as incorporating additional variables like dietary data or wearable sensor information, rather than broad statements like "larger datasets." Providing concrete examples would make the proposed extensions more meaningful and actionable.
